# Correction: ‘Strength of the “island rule” in birds is positively associated with absence of avian predators’ (2023), by Ponti *et al.*

**DOI:** 10.1098/rsbl.2023.0550

**Published:** 2023-12-13

**Authors:** Raquel Ponti, Claire Doutrelant, Rita Covas

**Affiliations:** ^1^ CIBIO-InBio, Centro de Investigação em Biodiversidade e Recursos Genéticos, Laboratório Associado, University of Porto, Campus Agrário de Vairão, Vairão, 4485-661 Portugal; ^2^ BIOPOLIS Program in Genomics, Biodiversity and Land Planning, CIBIO, Campus de Vairão, 4485-661 Vairão, Portugal; ^3^ Departamento de Biología Vegetal y Ecología, Universidad del País Vasco/Euskal Herriko Unibertsitatea (UPV/EHU), Leioa, Bizkaia, España; ^4^ CEFE, Univ Montpellier, CNRS, EPHE, IRD, Montpellier, France; ^5^ FitzPatrick Institute of African Ornithology, DST-NRF Centre of Excellence, University of Cape Town, Rondebosch 7701, South Africa

**Keywords:** ecological release, body mass, insularity, raptors, competitors

*Biol. Lett.* **19**: 20220536 (Published online 22 March 2023). (https://doi.org/10.1098/rsbl.2022.0536)

In the original paper, figure 1 showed model trends regarding the relationship between the body mass ratio (lnRR) of the island species and the body mass of the mainland species in the absence and presence of raptors (figure 1*a*), competitors belonging to the same genus (figure 1*b*) and competitors belonging to the same family (figure 1*c*). The trend lines and colour lines are correct in accordance with the models, but the colour of the points is incorrect. The three panels showed the points with the same colour scheme, corresponding to a random selection of the two colours for each point. We corrected the points with the colour corresponding to the absence and presence of predators or competitors. Blue points indicate the absence, and violet indicates the presence of predators and competitors.

**Figure 1.**
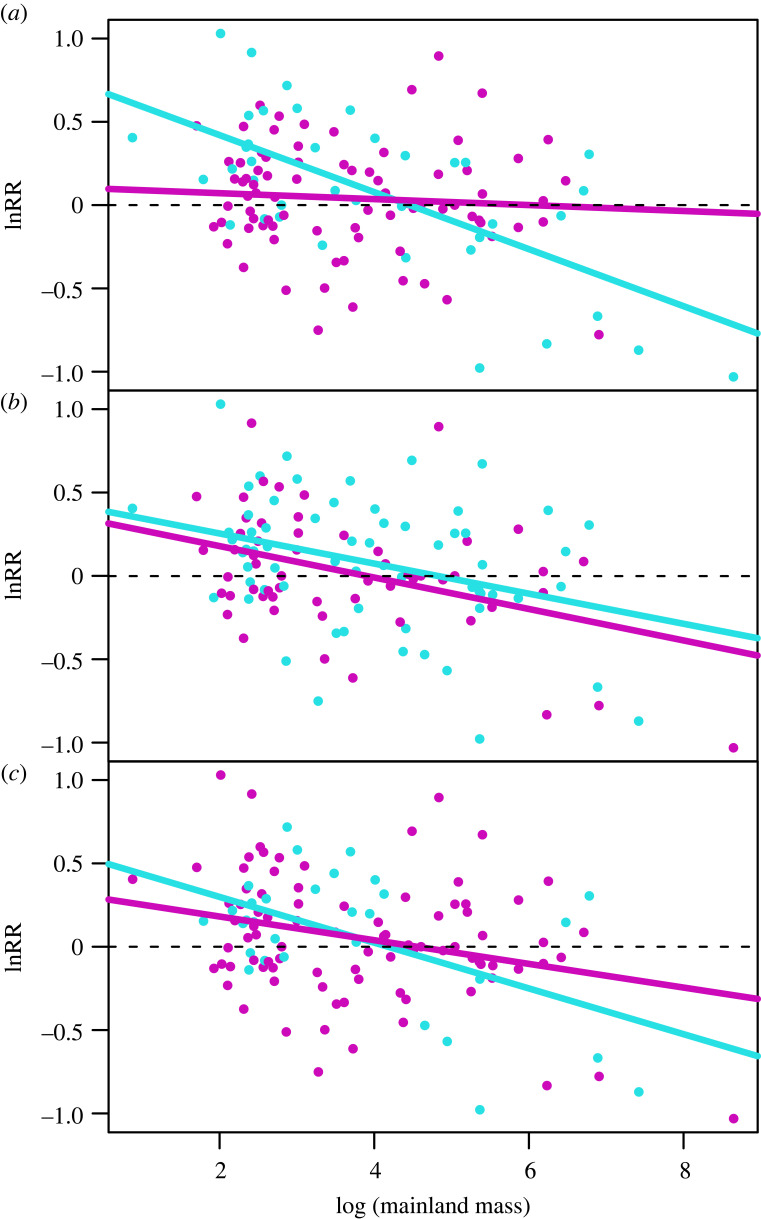
(*a*) Presence/absence of raptors changes the relationship between the body mass ratio (lnRR) and the body mass of the mainland species while (*b*) presence/absence of competitors belonging to the same genus does not and (*c*) presence/absence competitors belonging to the same family on islands has a marginal effect on this relationship. Points and lines in blue indicate absence and in violet presence of predators or competitors. The intercepts correspond to the binary models performed each with one variable (predation, competitors of the same genus or family). See models in electronic supplementary material, table S1 in appendix S4.

The correction does not affect the interpretation of the figure or the results, given that the lines correspond to the coefficients obtained in the models.

This has been amended on the publisher's website.

